# The Cytotoxic and Antimigratory Activity of Brazilin-Doxorubicin on MCF-7/HER2 Cells

**DOI:** 10.15171/apb.2018.059

**Published:** 2018-08-29

**Authors:** Riris Istighfari Jenie, Sri Handayani, Ratna Asmah Susidarti, Linar Zalinar Udin, Edy Meiyanto

**Affiliations:** ^1^Departement of Pharmaceutical Chemistry, Faculty of Pharmacy, Universitas Gadjah Mada, Indonesia.; ^2^Cancer Chemoprevention Research Center, Faculty of Pharmacy, Universitas Gadjah Mada, Indonesia.; ^3^Research Center for Chemistry, Indonesian Institute of Sciences (LIPI), Indonesia.

**Keywords:** Brazilin, Doxorubicin, Cytotoxic effect, Migration, MCF-7/HER2 cells

## Abstract

***Purpose:*** Breast cancer cells with overexpression of HER2 are known to be more aggressive, invasive, and resistant to chemotherapeutic agent. Brazilin, the major compound in the Caesalpinia sappan L. (CS) heartwood, has been studied for it's anticancer activity. The purpose of this study was to investigate the cytotoxic and antimigratory activity of brazilin (Bi) in combination with doxorubicin (Dox) on MCF-7/HER2 cells.

***Methods:*** Cytotoxic activities of Bi individually and in combination with Dox were examined by MTT assay. Synergistic effects were analyzed by combination index (CI). Apoptosis and cell cycle profiles were observed by using flow cytometry. Migrating and invading cells were observed by using a Boyden chamber assay. Levels of MMP2 and MMP9 activity were observed by using a gelatin zymography assay. Levels of HER2, Bcl-2, Rac1, and p120 protein expression were observed by using an immunoblotting assay.

***Results:*** The results of the MTT assay showed that Bi inhibited MCF-7/HER2 cell growth in a dose-dependent manner with an IC50 of 54 ± 3.7 µM. Furthermore, the combination of Bi and Dox showed a synergistic effect (CI <1). Flow cytometric analysis of Bi and its combination with Dox showed cellular accumulation in the G2/M phase and induction of apoptosis through suppression of Bcl-2 protein expression. In the Boyden chamber assay, gelatin zymography, and subsequent immunoblotting assay, the combination Bi and Dox inhibited migration, possibly through downregulation of MMP9, MMP2, HER2, Rac1, and p120 protein expression.

***Conclusion:*** We conclude that Bi enhanced cytotoxic activity of Dox and inhibited migration of MCF-7/HER2 cells. Therefore, we believe that it has strong potential to be developed for the treatment of metastatic breast cancer with HER2 overexpression.

## Introduction

Metastasis is the latest stage of cancer progression and is difficult to overcome.^1^ Metastasis is the process by which cancer cells leave the primary tumor and form secondary tumors at new sites. Several steps are involved in the metastasis process, including angiogenesis, loss of cell–cell adhesion, migration, invasion, and growth at the target organ site.^2^ Although much research has been focused on the discovery of agents that have a role in metastasis, the effectiveness of the agents remains limited^3^ and needs to be further explored.

Targeting drug discovery on the basis of molecular markers at every step of the metastatic cascade escalates the effectiveness of cancer treatment. ErbB2/HER2 (human epidermal growth factor receptor 2) is one of the important protein targets for cancer treatment. HER2 is a member of the epidermal growth factor receptor family that is overexpressed in many human cancers, especially breast cancer, and is related to invasiveness, drug resistance, and poor prognosis.^4^ Overexpression of HER2 induces proliferation, migration, and invasion of cancer cells through its downstream signaling pathway. Overexpression of this protein increases Src synthesis and activates Vav2, followed by activation of Ras homolog-Guanosine Triphosphate-ases (Rho-GTPases) such as Rac1, cell division cycle 42 (Cdc42), and Ras homolog A (RhoA) and modulation of cell migration.^5^ However, to activate HER2 signaling-induced migration, p120 catenin (p120) is needed as a Vav2 substrate.

Overexpression HER2 also has a role in increasing of the activation of matrix metalloproteases (MMPs), including MMP9 and MMP2.^5^ Invasive cancer cells secrete MMPs, which have the ability to degrade components of the basal matrix and the extracellular matrix (ECM), followed by invasion of cells to other sites. The expression and activation of MMPs have an important role in tumor growth and invasion.^2^ Many agents are studied for HER2-targeted therapy. Trastuzumab (Herceptin; Genentech, South San Francisco, CA) is an agent that competitively binds to the extracellular domain of HER2 and inhibits the HER2 signaling pathway.^6^ Flavonoids hesperetin and naringenin inhibit the HER2 activation pathway through the same action as lapatinib as a tyrosine kinase inhibitor.^7^ However, resistance of cancer cells to HER2-targeted agent was reported.^6,8^ It is important to investigate alternative agents that have a role in the HER2 pathway.

**Figure 1:**
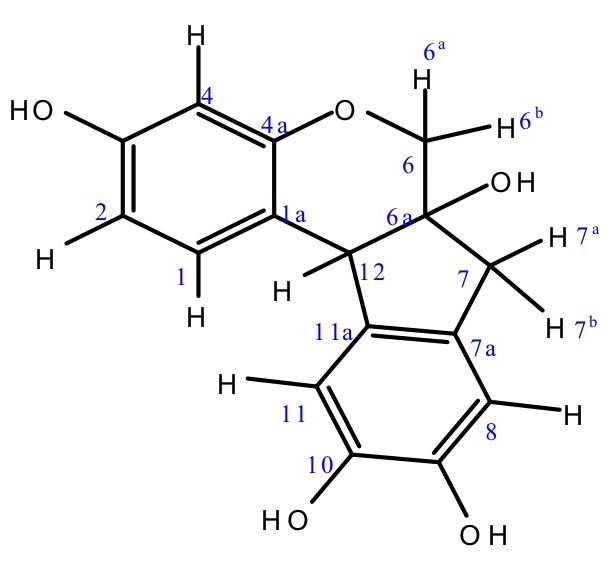
Chemical structure of brazilin

*Caesalpinia sappan* L. is a promising medicinal plant that is targeted at the metastasis stage. Several studies revealed the potential of this plant and its compounds, such as brazilin and brazilein, for use in cancer treatment.^9-11^ Brazilin (Figure 1) induces cell cycle arrest and inhibits MMP9 on cancer cells by suppressing nuclear factor (NF)-κB activation.^12^ Brazilein inhibits migration and invasion through suppression of Rac1 protein expression, as well as MMP2 and MMP9 activation and expression, on metastatic cancer cells.^13,14^ Because HER2 involves NF-κB, Rac1, and MMP protein upregulation, the potential cytotoxic and antimetastatic effect of brazilin on HER2 pathway and brazilin’s potency as a co-chemotherapeutic agent need to be explored.

Doxorubicin is a well-known chemotherapeutic agent for treatment of metastatic cancer. Unfortunately, on one hand, this agent causes many side effects, such as resistance of tumor cells and toxicity in normal cells. On the other hand, HER2-positive breast cancer cells cause a phosphoinositide 3-kinase (PI3K)-dependent activation of Akt and NF-κB. This mechanism is associated with increased resistance of the cells to multiple chemotherapeutic agents, including doxorubicin.^15,16^ To resolve these side effects, combination regimens have been developed to improve the effectiveness of cancer treatment.^17^ One of the benefits of combination therapy is reduction of the concentration of the chemotherapeutic agent, which may reduce its toxicity. Surprisingly, a low concentration of doxorubicin induces epithelial-to-mesenchymal transition (EMT) followed by an increase instead of inhibition of cancer metastasis.^18,19^ Therefore, brazilin has potential to be developed as a co-chemotherapeutic agent to counter doxorubicin-induced migration and invasion on HER2-overexpressing cancer cells. The goal of this study was to understand the role of the HER2 pathway as a mechanism of the cytotoxic and migration-inhibitory effect of brazilin and the combination of brazilin with doxorubicin on HER2 breast cancer cells (MCF-7/HER2).

## Materials and Methods

### Preparation of Samples

Doxorubicin was purchased from Sigma-Aldrich (St. Louis, MO). Dried heartwood powder of *Caesalpinia sappan* L. was obtained from B2P2TOOT (Tawangmangu, Indonesia). Dried powder was extracted in methanol by maceration to get the methanol extract. The methanol extract was diluted as 4:1 methanol/water and then partitioned with hexane. The aqueous layer was fractioned with ethyl acetate and concentrated with a vacuum rotary evaporator to get the ethyl acetate fraction. Brazilin (0.245 g) (Figure 1) was obtained by separation of ethyl acetate fractions using Sephadex G-15 column (Sigma-Aldrich) chromatography (15 × 7 cm) with gradient polarity of the mobile phase (CHCl_3_:MeOH) and was collected using thin-layer chromatography.

### Identification of Brazilin

#### High-Performance Liquid Chromatography

The profile of brazilin was obtained using a high-performance liquid chromatography (HPLC) instrument (Shimadzu LC-10; Shimadzu, Kyoto, Japan) under the following conditions: reversed-phase C-18 column (RP-18 LiChroCART 125-4; Millipore Sigma, Burlington, MA) with methanol/water (30:70 vol/vol) as a mobile phase with a flow rate of 1 ml/min.

#### Fourier Transform Infrared

Infrared spectra were obtained using the KBr pellet method with a Fourier transform infrared (FTIR) instrument (Spectrum 100; PerkinElmer, Waltham, MA). Infrared spectra of our brazilin showed a band of –OH bond at 3371 cm^−1^, a band of aliphatic C=C bond at 2928 cm^−1^, and a band of aromatic C=C bond at 1610 cm^−1^. The absence of carbonyl group (C=O) spectra at 1700 cm^−1^ on brazilin is the main difference between brazilin and brazilein.

#### Liquid Chromatography-Mass Spectrometry

Liquid chromatography-mass spectrometry (LC-MS) (Mariner Biospectrometry workstation [McKinley Scientific, Sparta, NJ], Hitachi L-6200 [Hitachi, Tokyo, Japan]) was performed using a Supelco reversed-phase C-18 column (250 mm × 2 mm, 5 μm; Sigma-Aldrich) with an electrospray ionization (ESI) system (positive ion mode). The ESI mass spectrum was presented at 287 mass-to-charge ratio, corresponding to the [M+H]^+^ of brazilin (molecular weight, 286 g/mol).

#### H-NMR and C-NMR

Analysis was also carried out using nuclear magnetic resonance (NMR) spectrometry (JNM-ECA 500 spectrometer; JEOL, Tokyo, Japan) with proton nuclear magnetic resonance (^1^H-NMR) and carbon nuclear magnetic resonance (C-NMR). The NMR data of the C-isolate showed ^1^H-NMR (500 MHz, in acetone-d_6_) 7.19 (^1^H, d, *J* = 8.43 Hz, H-1), 6.49 (^1^H, dd, *J =* 2.6 and 8.43 Hz, H-2), 6.31 (^1^H, d, *J =* 2.6 Hz, H-4), 3.94 (^1^H, d, *J =* 11.03 Hz, H-6^a^), 3.71 (^1^H, d, *J =* 11.03 Hz, H-6^b^), 3.01 (^1^H, d, *J =* 15.6 Hz, H-7^a^), 2.81 (^1^H, d, *J =* 15.6 Hz, H-7^b^), 6.76 (^1^H, s, H-8), 6.65 (^1^H, s, H-11), and 3.97 (^1^H, s, H-12); and ^13^C-NMR (125 MHz, in acetone-d_6_) 132.0 (C-1), 109.6 (C-2), 155.5 (C-3), 104,0 (C-4), 157.6 (C-4a), 70.8 (C-6), 77.8 (C-6a), 42.9 (C-7), 131.5 (C-7a), 112.7 (C-8), 144.8 (C-9), 144.6 (C-10), 112.4 (C-11), 137.4 (C-11a), and 51.1 (C-12). Based on comparison of the HPLC, FTIR, LC-MS, and NMR data, our findings for brazilin were similar to previously reported data.^20^

#### Cell Culture

The MCF-7/HER2 and MCF-7/empty vector (MCF-7/Mock) cell lines were kindly provided by Prof. Yoshio Inouye, mediated by Prof. Dr. Masashi Kawaichi (Nara Institute of Science and Technology). These cells were cultured in Dulbecco’s modified Eagle’s medium (Thermo Fisher Scientific, Waltham, MA) with 10% fetal bovine serum (FBS) (Thermo Fisher Scientific), 1.5% penicillin-streptomycin (Thermo Fisher Scientific), and 0.5% amphotericin B (Thermo Fisher Scientific).

#### Cytotoxic Assay with Individual Samples and Combination Samples

The cells (1 × 10^4^/well) in 96-well plates were treated with various concentrations of the different treatment groups. After 24-h incubation, culture medium was removed and cells were washed in phosphate-buffered saline (PBS) (Sigma-Aldrich). Then, cells were incubated for 4 h with 100 µL of culture medium and 10 µL of 3-(4,5-dimethylthiazol-2-yl)-2,5-diphenyltetrazolium bromide (MTT) (Sigma-Aldrich) with 5 mg/mL in every well. The MTT reaction was stopped using sodium dodecyl sulfate (SDS) reagent (10% SDS in 0.01 M HCl; Millipore Sigma) and incubated overnight. The absorbance was measured with a microplate reader (Bio-Rad Laboratories, Hercules, CA) at 595 nm. The combination index (CI) was calculated using CompuSyn software (version 1.0; ComboSyn, Paramus, NJ).

#### Cell Cycle Distribution

A propidium iodide (PI) staining kit (BD Biosciences, San Jose, CA) was used to analyze DNA content. Cells were seeded into 24-well plates with 5 × 10^4^ cells/well and treated with various concentrations of samples alone and in combination. After a 24-h treatment, cells were harvested, fixed with 70% ethanol, labeled with PI/RNase stain (2 µg/mL), and incubated at room temperature (RT) in the dark for 10 minutes. The DNA content was analyzed using flow cytometry (BD Biosciences) and Flowing software (version 2.5.1; Cell Imaging Core, Turku Centre for Biotechnology, Turku, Finland).

#### Apoptosis Detection

Populations of apoptotic cells were determined by PI-annexin V assay (Annexin V-FITC Apoptosis Detection Kit; Roche Mannheim, Germany). Cells (5 × 10^4^/well) were seeded into a 24-well plate and treated with various concentrations of samples, alone and in combination. After a 24-h treatment, cells were harvested, added to 1× binding buffer, labeled with PI-annexin V, and incubated at RT in the dark for 5 minutes. Then, the cell suspension was analyzed using flow cytometry (BD Biosciences).

#### Migration and Invasion Assay

Cell migration and invasion were assayed in accordance with CytoSelect^TM^ cell migration and invasion assay protocol (Cell Biolabs, San Diego, CA). Cells were serum-starved for 24 h, harvested, and suspended in 0.5% FBS/DMEM. Cells (3 × 10^5^ cells/well) were seeded into the upper compartment of an insert chamber with or without samples on both migration and invasion compartments. The 10% FBS/DMEM medium was placed in the lower chamber. After a 24-h incubation at 37°C, nonmigrating cells on the upper side of the membrane were wiped off the upper compartment, and migrating cells on the lower side of the membrane were stained using the CytoSelect^TM^ staining kit for 10 min at RT. After being gently washed and dried, cells were dissolved with extraction solution. The absorbance was measured using a microplate reader (SH-1000; Corona Electric Co., Hitachinaka, Japan) at 560 nm.

#### Gelatin Zymography

Secretion of MMP9 and MMP2 in the medium was assayed by gelatin zymography. Cells (1 × 10^6^) were seeded into each well of a 6-well plate and incubated at 37°C in a CO_2_ incubator for 24 h. Cells were incubated with a quarter of the half maximal inhibitory concentration (¼ IC_50_) of samples, alone and in combination, in serum-free medium for 24 h. The medium was collected and subjected to polyacrylamide gel electrophoresis (PAGE) on 10% SDS-PAGE gel containing 0.1% gelatin and run in the SDS running buffer. The gels were washed in renaturing solution containing 2.5% Triton X-100 for 30 minutes, then incubated with incubation buffer (50 mM Tris-HCl, 150 mM NaCl, 10 mM CaCl_2_) for 20 h at 37°C. The gels were stained using 0.5% Coomassie Brilliant Blue and incubated for 30 min at RT and destained with destaining solution (10% v/v methanol and 5% v/v acetic acid). Gels were then scanned and documented.

#### Immunoblotting Assay

Cells (1 × 10^6^) were seeded into a 10-cm culture dish and incubated at 37°C in a CO_2_ incubator for 24 h. Cells were incubated with ¼ IC_50_ of samples, alone and in combination, for 24 h. Cells were collected with radioimmunoprecipitation assay (RIPA) buffer (25 mM Tris-HCl, pH 7.6, 150 mM NaCl, 1% Nonidet P-40, 1% deoxycholic acid-Na, 0.1% SDS, protease and phosphatase inhibitor cocktail). Protein concentrations were determined using the Bradford assay method, measured using a microplate reader (SH-1000; Corona Electric Co.). Then, samples were separated by electrophoresis on 7–15% SDS-PAGE gels and electrotransferred onto PVDF transfer membranes (Immobilon; Millipore Sigma). After being blocked with 1× NET gelatin buffer, the membranes were probed with antibodies for Rac1 (ab33186; Abcam, Cambridge, UK), HER2 (sc-52439), p120 (sc-13957), Bcl-2 (sc-7382), and β-actin (sc-47778; Santa Cruz Biotechnology, Dallas, TX) and then exposed to horseradish peroxidase-conjugated secondary antimouse (sc-2031; Santa Cruz Biotechnology) or antirabbit (7074P2, Cell Signaling Technology, Danvers, MA) antibodies. Protein expression was detected using an Amersham enhanced chemiluminescence system (GE Healthcare Life Sciences, Marlborough, MA).

#### Immunofluorescence Microscopy

Cells (5 × 10^4^) were seeded onto coverslips in 24-well plates and incubated at 37°C in a CO_2_ incubator for 24 h. Cells were incubated with a half of IC_50_ (**½** IC_50_) of samples, alone and in combination, for 24 h. Cells were washed with PBS, and after fixation with 70% cold ethanol and blocking with 1% bovine serum albumin (BSA), they were incubated with primary antibody for HER2 (sc-52439) followed by Alexa Fluor 488 secondary antibody. Then, cells were washed and incubated with 4′,6-diamidino-2-phenylindole (DAPI). Coverslips were moved into object glass and analyzed using a fluorescence microscope (Zeiss MC 80; Carl Zeiss Microscopy, Jena, Germany) equipped with blue argon (for DAPI) and green argon (for Alexa Fluor 488) lasers.

#### Statistical Analysis

Statistical analysis was performed using Student’s *t* test (Excel 2013 software; Microsoft, Redmond, WA). *P* values less than 0.05 were considered significant. Effects of combinations on growth inhibition were analyzed using the CI equation developed by Reynolds and Maurer.^21^ Gelatin zymography results were calculated by using ImageJ software (National Institutes of Health, Bethesda, MD).

## Results and Discussion

### Cytotoxic Assay of Samples Alone and in Combination

**Figure 2:**
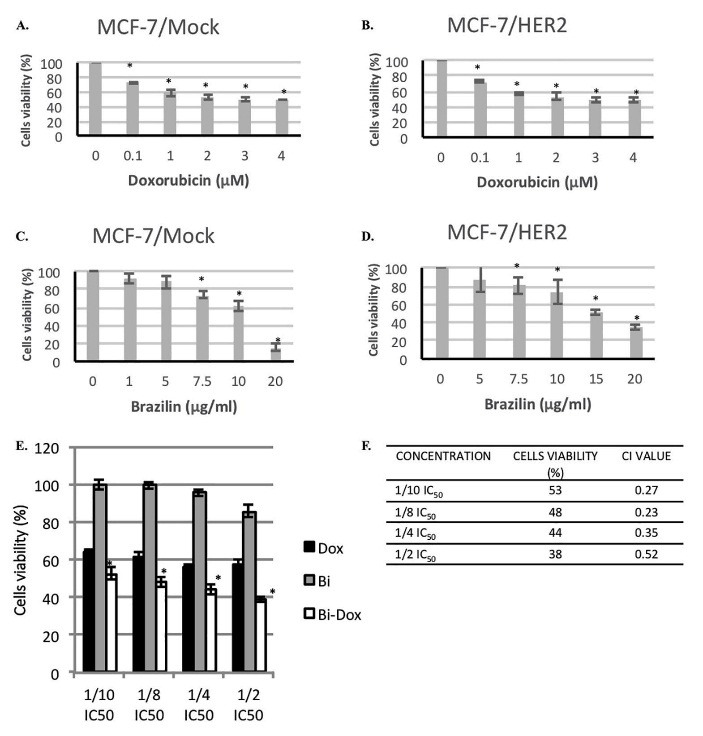
Cytotoxic activity of treatment with brazilin alone and its combination with doxorubicin on MCF-7/HER2 cells. Effects of treatment of MCF-7/Mock (A) and MCF-7/HER2 (B) with doxorubicin alone and treatment of MCF-7/Mock (C) and MCF-7/HER2 (D) with brazilin alone are shown. The combination of brazilin and doxorubicin (1/10-1/2 IC_50_) (E) and the combination index value of the combination of brazilin and doxorubicin (F) effects on MCF-7/HER2 cells are also depicted. Cells were treated with various concentrations of samples for 24 h before assessment by MTT assay. Error bar represents standard deviation (n = 3, *P < 0.05 by Student’s *t* test)

Brazilin was reported to have anticancer activity by inducing cell cycle arrest.^12^ Therefore, we performed cytotoxic assays to confirm the potency of brazilin as an anticancer agent. The cytotoxic effect of brazilin and doxorubicin was measured by MTT assay. After 24-hour incubation, doxorubicin inhibited MCF-7/Mock and MCF-7/HER2 cell growth with similar IC_50_ values (3 µM) (Figures 2A and 2B), whereas brazilin inhibited MCF-7/Mock and MCF-7/HER2 cell growth in a dose-dependent manner with IC_50_ values of 44 ± 2.4 µM and 54 ± 3.7 µM, respectively (Figures 2C and 2D). These results show that brazilin possessed moderate cytotoxic activity but that it has potential to be developed as a co-chemotherapeutic agent.

Next, to confirm whether brazilin enhanced the cytotoxic activity of doxorubicin, we analyzed the synergistic combination by using the CI. Combinations of 1/10, **⅛, ¼,** and **½** IC_50_ of brazilin/doxorubicin showed a synergistic effect on inhibition of MCF-7/HER2 cell growth (CI <1) (Figures 2E and 2F). The combination of **½** IC_50_ brazilin/doxorubicin inhibited cell viability up to 62% compared with untreated cells. The findings regarding the combination of brazilin and doxorubicin indicated promise as a compound for HER2-positive breast cancer treatment. The synergistic cytotoxic activity may occur as a result of inhibition of cell cycle modulation or apoptosis induction. Accordingly, we observed the effect of brazilin and its combination with doxorubicin on cell cycle modulation and apoptosis in further experiments.

### Cell Cycle and Apoptosis Modulation

**Figure 3:**
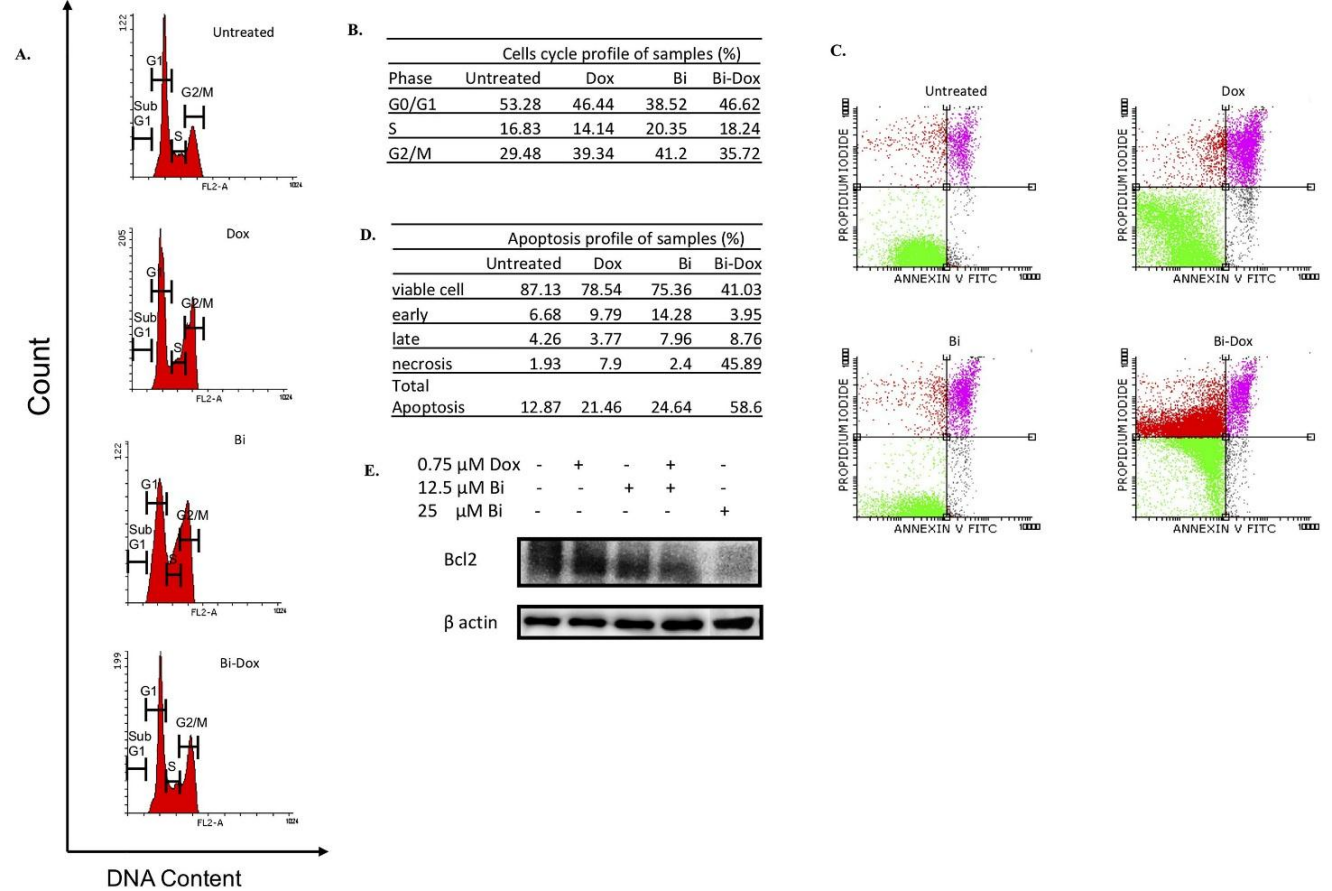
The effect of treatment with brazilin alone and its combination with doxorubicin on MCF-7/HER2 cell cycle profiles and apoptosis. Cells were treated with vehicle (untreated), 1.5 µM (**½** IC_50_) doxorubicin, 25 µM (**½** IC_50_) brazilin, and the combination of **½** IC_50_ brazilin and **½** IC_50_ doxorubicin for 24 h, then stained with PI/RNase for cell cycle analysis (A) or with PI-annexin V for apoptosis analysis (C). The analysis of cell cycle and apoptosis were conducted by using flow cytometry as described in the Materials and Methods; and quantified by using Flowing software (B and D). Cells were treated with brazilin alone and in combination with doxorubicin for 24 h, and the Bcl-2 protein levels (E) were observed by immunoblotting assay

Flow cytometric analysis for cell cycle showed that a single treatment of **½** IC_50_ brazilin or **½** IC_50_ doxorubicin caused a G_2_/M phase accumulation compared with untreated cells (Figures 3A and 3B). Combination treatment with **½** IC_50_ brazilin and **½** IC_50_ doxorubicin induced G_2_/M phase accumulation compared with either treatment alone (Figures 3A and 3B). Moreover, flow cytometric analysis for apoptosis showed that after 24-h incubation, treatment with either **½** IC_50_ doxorubicin or **½** IC_50_ brazilin alone induced apoptosis up to 9% and 12%, respectively, compared with untreated cells (Figures 3C and 3D). Combination of **½** IC_50_ brazilin and **½** IC_50_ doxorubicin increased necrosis rather than apoptosis (Figures 3C and 3D). We hypothesized that the necrosis event occurred after apoptosis induction. In *in vitro* studies, apoptosis leading to necrosis is the normal phenomenon of cell death owing to the absence of phagocytic cells.^22^ Next, to confirm our hypothesis, we observed the level of Bcl-2 protein expression. The result showed that brazilin alone and in combination with doxorubicin decreased the level of Bcl-2 protein expression (Figure 3E). Therefore, combination of brazilin and doxorubicin inhibited proliferation possibly by inducing apoptosis and cellular accumulation in G_2_/M phase.

### Inhibition of Migration and Invasion

**Figure 4:**
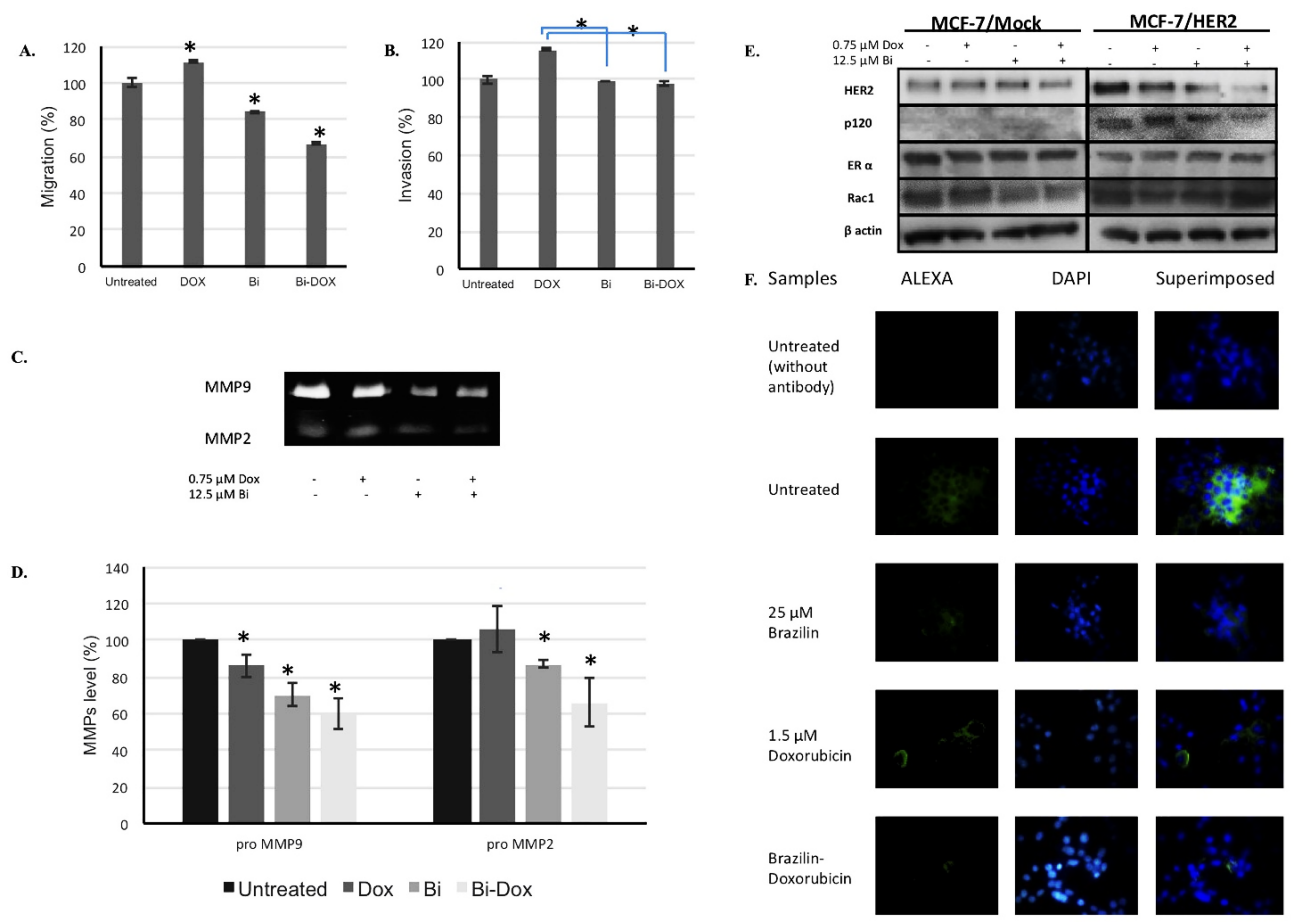
The effect of treatment with brazilin alone and its combination with doxorubicin on MCF-7/HER2 cell migration and invasion. Cells were incubated with **¼** IC_50_ of brazilin or doxorubicin, alone and in combination, for 24 h in low serum concentration. Then, migration (A) and invasion (B) of cells under the chamber were measured using the migration and invasion assay. Cells were treated with **¼** IC_50_ of brazilin or doxorubicin, alone and in combination, for 24 h. Then, the levels of the MMP protein bands were observed by gelatin zymography (C) and calculated by using ImageJ software (D). The levels of the HER2, p120, ERα, and Rac1 protein bands were observed by immunoblotting assay (E). Cells were treated with **½** IC_50_ of brazilin or doxorubicin, alone and in combination, for 24 h and were observed according to the immunofluorescence method to visualize the alteration of HER2 expression resulting from treatment (F). Error bar represents standard deviation (n = 3, *P < 0.05 by Student’s *t* test)

To study whether the combination of brazilin and doxorubicin had an antimetastatic effect on MCF-7/HER2 cells, we first tested the effect of each agent alone and in combination as **¼** IC_50_ of brazilin/doxorubicin by migration and invasion assay. On one hand, the result showed that treatment with 0.75 µM doxorubicin alone increased migration and invasion of MCF-7/HER2 cells up to 11% and 16%, respectively. On the other hand, treatment with 12.5 µM brazilin alone, inhibited migration (up to 16%) but not invasion compared with untreated cells. Interestingly, the addition of brazilin to doxorubicin treatment inhibited migration and invasion up to 44% and 18%, respectively, compared with doxorubicin alone (Figures 4A and 4B).

### Inhibition of MMP2, MMP9, HER2, p120, and Rac1 Protein Expression

Metastasis is a set of complex processes comprising internal and external molecular events. The high expression of proteinases such as MMP9 and MMP2 in the microenvironment of cancer cells is an example of external molecular events known to be involved in the degradation of the ECM and to play a critical role in tumor invasion and metastasis.^23^ To understand the molecular mechanism that plays a role in inhibition of MCF-7/HER2 cell migration and invasion as a result of the treatments, we thus tested the effect of brazilin and its combination with doxorubicin on alteration of MMP2 and MMP9 protein expression according to gelatinolytic activity by using gelatin zymography. The results indicated that** ¼** IC_50_ brazilin alone and in combination with doxorubicin decreased MMP2 and MMP9 protein levels on MCF-7/HER2 cells (Figures 4C and 4D).

The HER2 pathway has an important role in the migration and invasion of cancer cells. In the present study, we observed the effect of brazilin and its combination with doxorubicin on modulation of HER2 protein expression on MCF-7/HER2 and MCF-7/Mock cells. The results showed that treatment with brazilin alone decreased HER2 protein levels (Figure 4E). This result was confirmed with immunofluorescence data that showed a downtrend of protein expression by the combination of brazilin and doxorubicin (Figure 4F). We also observed the effect of the combination of brazilin and doxorubicin on modulation of p120 and Rac1 proteins that have a role in HER2 overexpression and cell migration. The combination of brazilin and doxorubicin indicated a downtrend of p120 and Rac1 protein levels compared with untreated cells (Figure 4E). Estrogen receptor-α (ERα) is upregulated during HER2 therapy.^24^ Then, we also checked the effect of brazilin treatment on ERα protein levels. The results showed that treatment of brazilin and its combination with doxorubicin did not affect ERα protein expression (Figure 4E). The combination of brazilin and doxorubicin showed a downtrend of HER2, p120, and Rac1 protein levels compared with untreated cells (Figures 4E and 4F).

Migration and invasion are the basic metastatic stages of breast cancer. Importantly, overexpression of HER2 protein worsens the prognosis of metastatic cancer.^25^ This study shows that the isoflavone brazilin has synergistic cytotoxic effects when combined with doxorubicin against MCF-7/Mock as well as MCF-7/HER2 cells. The flavonoids apigenin, hesperetin, and naringenin sensitize HER2-positive breast cancer cells, leading to cell death.^7,26^ Wighteone, an isoflavone derived from *Erythrina suberosa*, inhibits the proliferation of MCF-7 HER2-positive breast cancer cells.^27^ Combination of polyphenols, including flavonoids, with other anticancer drugs increases the antitumor effects more than treatment using only one of the compounds.^17^ The present study reveals the potency of brazilin as a co-chemotherapeutic agent for treatment of HER2-overexpressing breast cancer.

In order to confirm the mechanism that has a role in the synergistic cytotoxic effect of brazilin and doxorubicin on MCF-7/HER2 cells, studies of cell cycle modulation and apoptosis need to be done. We found that brazilin, doxorubicin, and their combination induce G_2_/M accumulation (Figures 3A and 3B). On one hand, doxorubicin induces G_2_/M arrest through its action as a type II topoisomerase inhibitor.^28^ On the other hand, this study also confirms the finding of Kim *et al.*^12^ that brazilin causes G_2_/M arrest on U266 myeloma cells. Several isoflavones, such as genistein and DW532, induce G_2_/M accumulation through binding on tubulin and leading to depolymerization of microtubules.^29,30^ Because brazilin has an isoflavone structure, the effect of G_2_/M accumulation by brazilin may travel the same pathway. Thus, brazilin and doxorubicin synergistically induce G_2_/M arrest through different pathways.

This study also reveals that brazilin and its combination with doxorubicin induces apoptosis on MCF-7/HER2 cells by decreasing of Bcl-2 protein expression (Fig. 3E). Because the apoptotic mechanism of doxorubicin induces apoptosis through the FAS/FAS ligand,^31^ the decrease in Bcl-2 seen in this study may be mainly attributable to brazilin. Decreasing Bcl-2 expression is followed by activation of caspases, leading to apoptosis.^32^ Brazilin induces apoptosis through a caspase-dependent pathway.^33^ HER2 overexpression activates the NF-κB transcription factor, which is involved with transcription of many genes, including *Bcl2.*^34^ Jeon *et al*. reported that brazilin inhibits activation of NF-κB.^35^ The flavonoid curcumin and its analog sensitized doxorubicin through inhibition of HER2 and activation of NF-κB.^36^ Inactivation of NF-κB via the HER2 pathway may have a role in the induction of apoptosis by brazilin.

Migration and invasion are the important parts of the metastatic process.^2^ This study reveals the inhibition of cell migration by brazilin (Figures 4A and 4B). Previous studies revealed antimigratory effects of the flavonoids brazilein and baicalein.^13,14,37^ Secretion of MMP protein in the tumor microenvironment has a role in supporting migration and invasion of cancer cells through ECM degradation.^23^ Our study shows the downregulation of MMP2 and MMP9 protein levels by treatment with brazilin alone and its combination with doxorubicin on HER2-overexpressing cells (Figures 4C and 4D). These data are in line with previous studies which showed that brazilein inhibits MMP2 on MDA-MB-231 cells and that its combination with cisplatin showed downregulation of MMP9 on 4T1 cells.^13,14^ Other flavonoids, such as 7,7″-dimethoxyagastisflavone, luteolin, quercetin, and a curcumin analog (potassium pentagamavunon-0, K PGV-0), inhibit metastasis through suppression of MMP secretion.^38-40^ Because NF-κB transcripts MMP protein^41^ and HER2 protein has a role on NF-κB protein activation,^42^ we drew an inference about the effect of inhibitory effects on migration and invasion by brazilin and its combination with doxorubicin on MCF-7/HER2 cells probably being related to the HER2/NF-κB pathway.

Furthermore, we confirmed our hypothesis that brazilin and its combination with doxorubicin would suppress HER2 protein expression (Figures 4E and 44). Many studies found the HER2-inhibitory effect of flavonoids on cancer cells. Berberine, apigenin, and amentoflavone inhibit cell growth by downregulating HER2 protein expression.^43-45^ Other proteins that are well known as key regulators of cell migration through the HER2 pathway are Rac1 and p120 catenin protein.^5^ Rac1 expression induces migration and increases the resistance mechanism of anti-HER2 therapies.^46^ This study shows downregulation of Rac1 protein expression by brazilin. Curcumin and wogonin inhibit cell migration by suppressing Rac1 protein expression.^47,48^ Brazilein and its combination with cisplatin were revealed to downregulate Rac1 but not p120 protein expression on 4T1, a triple-negative breast cancer cell.^14^ Interestingly, this study proves that the combination of brazilin with doxorubicin downregulates HER2, Rac1, and p120 protein expression on HER2-overexpressing cancer cells (Figure 4E). The expression of p120 is needed for migration and invasion of HER2-positive breast cancer cells.^5^ However, the mechanism that has a role in inhibition of p120 expression by brazilin was not previously clearly understood. It probably is associated with its action on inactivation of NF-κB/Snail. Researchers in a previous study reported that apigenin inhibits EMT via inhibiting the NF-κB/Snail pathway.^49^ Expression of Snail mediated by NF-κB activation increases splicing of the 120 kD isoform of p120 catenin.^50^ Snail is known to have an important role on EMT induced by doxorubicin. On one hand, we hypothesized that brazilin-sensitized migration cells may increase via doxorubicin through this mechanism. On the other hand, Johnson *et al.*^5^ reported that activation of Rho-GTPases, including Rac1, correlate with p120 levels in HER2-expressing cells. Thus, brazilin may suppress not only Rac1 expression but also its activation. However, further investigation is needed.

Cross-talk between ERα and HER2 induced HER2-resistant cancer cells.^51^ The presence of ERα may interfere with agents that target the HER2 receptor.^24^ To obtain additional data, we confirmed that brazilin and its combination with doxorubicin did not affect ERα expression. This means that suppression of HER2 expression by brazilin may not interfere with expression of ERα. Nevertheless, further studies are needed to confirm the mechanism that has a role in cytotoxic and migration-inhibitory effects of brazilin in combination with doxorubicin on HER2-overexpressing breast cancer cells. Brazilin has potential to be developed as a co-chemotherapeutic agent for metastatic cancer with HER2 overexpression.

## Conclusion

This study shows that brazilin and doxorubicin work synergistically in inducing cytotoxicity in MCF-7/HER-2 cells, as shown by the CI value less than 1. The mechanisms involved were cell cycle arrest at the G_2_/M phase and apoptosis induction by suppressing Bcl-2 expression. Moreover, we found that brazilin inhibited migration and invasion of MCF-7/HER-2 cells, whereas doxorubicin increased it. The mechanism involved was downregulation of the expression of HER2, p120, MMP2, and MMP9. Thus, brazilin has potential to be developed in combination with chemotherapeutic agents to increase cytotoxicity and to inhibit migration and invasion toward HER2-overexpressing breast cancer cells.

## Acknowledgments

We thank to PUPT 2015-2016 from Indonesian Ministry of Research and Technology and High Education for the project grant. We also thank to Dr. Ahmad Darmawan for the NMR identification and Dra. *Puspa* Dewi Narrij Lotulung, M. Eng from Indonesian Institute of Sciences (LIPI) for LC/MS identification and thank Prof. Masashi Kawaichi, MD, Ph.D. from Nara Institute of Science and Technology, Japan, for providing the cell lines and for the technical assistance in this project.

Some parts of the data in this publication were used in the thesis dissertation of Dr. Sri Handayani

## Ethical Issues

Not applicable.

## Conflict of Interest

We declare that we have no conflict of interest.
